# Buprenorphine/naloxone initiation and referral as a quality improvement intervention for patients who live with opioid use disorder: quantitative evaluation of provincial spread to 107 rural and urban Alberta emergency departments

**DOI:** 10.1007/s43678-023-00520-3

**Published:** 2023-05-28

**Authors:** Kayla D. Stone, Ken Scott, Brian R. Holroyd, Eddy Lang, Karen Yee, Niloofar Taghizadeh, Janjeevan Deol, Kathryn Dong, Josh Fanaeian, Monty Ghosh, Keysha Low, Marshall Ross, Robert Tanguay, Peter Faris, Nathaniel Day, Patrick McLane

**Affiliations:** 1 Department of Psychiatry, University of Calgary, Calgary, AB Canada; 2 Emergency Strategic Clinical Network™, Alberta Health Services, Edmonton, AB Canada; 3 Department of Emergency Medicine, University of Alberta, Edmonton, AB Canada; 4 Department of Emergency Medicine, University of Calgary, Calgary, AB Canada; 5 Data and Analytics (DIMR), Alberta Health Services, Edmonton, AB Canada; 6 Department of Medicine, University of Calgary, Calgary, AB Canada; 7 Department of General Internal Medicine, University of Alberta, Edmonton, AB Canada; 8 Department of Surgery, University of Calgary, Calgary, AB Canada; 9 Virtual Opioid Dependency Program, Alberta Health Services, Edmonton, AB Canada

**Keywords:** Opioid use disorder, Opioid agonist treatment, Addiction, Quality improvement, Buprenorphine/naloxone, Trouble lié à l’utilisation d’opioïdes, Traitement par agonistes opioïdes, Toxicomanie, Amélioration de la qualité, Buprénorphine/naloxone

## Abstract

**Objectives:**

Opioid use disorder is a major public health concern that accounts for a high number of potential years of life lost. Buprenorphine/naloxone is a recommended treatment for opioid use disorder that can be started in the emergency department (ED). We developed an ED-based program to initiate buprenorphine/naloxone for eligible patients who live with opioid use disorder, and to provide unscheduled, next-day follow-up referrals to an opioid use disorder treatment clinic (in person or virtual) for continuing patient care throughout Alberta.

**Methods:**

In this quality improvement initiative, we supported local ED teams to offer buprenorphine/naloxone to eligible patients presenting to the ED with suspected opioid use disorder and refer these patients for follow-up care. Process, outcome, and balancing measures were evaluated over the first 2 years of the initiative (May 15, 2018–May 15, 2020).

**Results:**

The program was implemented at 107 sites across Alberta during our evaluation period. Buprenorphine/naloxone initiations in the ED increased post-intervention at most sites with baseline data available (11 of 13), and most patients (67%) continued to fill an opioid agonist prescription at 180 days post-ED visit. Of the 572 referrals recorded at clinics, 271 (47%) attended their first follow-up visit. Safety events were reported in ten initiations and were all categorized as no harm to minimal harm.

**Conclusions:**

A standardized provincial approach to initiating buprenorphine/naloxone in the ED for patients living with opioid use disorder was spread to 107 sites with dedicated program support staff and adjustment to local contexts. Similar quality improvement approaches may benefit other jurisdictions.

**Supplementary Information:**

The online version contains supplementary material available at 10.1007/s43678-023-00520-3.

## Abstrait

**Objectifs:**

Le trouble lié à la consommation d’opioïdes est une préoccupation majeure en santé publique qui explique le nombre élevé d’années potentielles de vie perdues. La buprénorphine/naloxone est un traitement recommandé pour le trouble lié à l’utilisation d’opioïdes qui peut être commencé au service des urgences (SU). Nous avons mis au point un programme axé sur les urgences pour commencer la buprénorphine/naloxone pour les patients éligibles qui vivent avec un trouble lié à l’utilisation d’opioïdes, et pour fournir suivis des cas référés le jour suivant vers une clinique de soins des troubles liés à l’utilisation d’opioïdes (sur place ou virtuelle) pour les soins continus aux patients partout en Alberta.

**Méthodes:**

Dans le cadre de cette initiative d’amélioration de la qualité, nous avons aidé les équipes locales de SU à offrir la buprénorphine/naloxone aux patients admissibles qui se présentent à la SU avec un trouble présumé de consommation d’opioïdes et à les diriger vers des soins de suivi. Le processus, les résultats et les mesures d’équilibre ont été évalués au cours des deux premières années de l’initiative (du 15 mai 2018 au 15 mai 2020).

**Résultats:**

Le programme a été mis en œuvre dans 107 sites en Alberta pendant notre période d’évaluation. Les initiations à la buprénorphine/naloxone à l’urgence ont augmenté après l’intervention dans la plus grande partie de sites pour lesquels des données de référence étaient disponibles (11 sur 13), et la plupart des patients (67 %) ont continué de remplir une ordonnance d’agonistes opioïdes 180 jours après la visite à l’urgence. Sur les 572 renvois enregistrés aux cliniques, 271 (47 %) ont assisté à leur première visite de suivi. Des événements liés à la sécurité ont été signalés dans 10 initiatives et ont tous été classés comme n’ayant causé aucun conséquences à des conséquences minimes.

**Conclusions:**

Une approche provinciale standardisé de lancement de la buprénorphine/naloxone à l’urgence pour les patients atteints d’un trouble lié à la consommation d’opioïdes a été diffusée à 107 sites à l’aide de soutien aux programmes spécialisé et des ajustements aux contextes locaux. Des approches semblables d’amélioration de la qualité pourraient profiter à d’autres juridictions.

## Clinician’s capsule

**Table:** 

***What is known about the topic?***
Opioid agonist treatment can be initiated in emergency departments.
***What did this study ask?***
This study evaluated the expansion of an opioid agonist treatment initiation program across Alberta EDs, patient demographics, and outcomes.
***What did this study find?***
Most sites offered more opioid agonist treatment after program initiation, and most patients initiated on treatment continued in treatment.
***Why does this study matter to clinicians?***
Other jurisdictions may benefit from standardized programs to support opioid agonist treatment across multiple sites.

## Introduction

Between January 2016 and September 2022, 34,455 Canadians had opioid-related deaths [1], with an increase during the COVID-19 pandemic [2]. Opioid agonist treatment significantly reduces mortality in persons with opioid use disorder [3, 4]. Current Canadian guidelines strongly recommend buprenorphine/naloxone as a first-line treatment [5–7]. We examine the process, outcome, and balancing measures related to buprenorphine/naloxone initiation in 107 emergency department and urgent care (ED) sites.

Since 2016, opioid-related ED visits in this province have risen, from 7816 in 2016 to 10,368 in 2020 when our evaluation data was collected [8]. Fentanyl and its derivatives were involved in 89% of opioid-related deaths in 2020, with carfentanil involved in 10% [9]. Programs that focus on initiating buprenorphine/naloxone in EDs have been developed across Canada and the USA [10–14]. Benefits include continuity of care for patients [10] and increased outpatient treatments [13]. Our study evaluates provincial expansion of one such program and is of value, as current literature is largely limited to studies on single EDs [15]. Our goal for the phase of the project reported in this manuscript was to expand the program provincially, with the intent of including all ED sites across the province. The specific aim was to increase buprenorphine/naloxone initiations in EDs in Alberta.

## Methods

### Study design and time period

Administrative data were used to evaluate the quality improvement program during a pre-specified evaluation period from May 15, 2018 to May 15, 2020. Data collection details are provided in Online Resource 1. Exemption from ethics review was received from the University of Alberta Health Research Ethics Board, which determined that the work was an evaluation of quality improvement.

### Population

An expert working group consisting of addiction and emergency medicine specialists established patient treatment criteria: (1) suspicion of opioid use disorder and (2) patient willing to engage in buprenorphine/naloxone treatment. Exclusion criteria were (1) allergy to buprenorphine/naloxone, (2) being admitted for medical/psychiatric concern, (3) severe liver dysfunction, (4) using methadone or buprenorphine/naloxone, or (5) sedative/depressive impairment or intoxication. Pregnant patients were included and consultation with an addiction or obstetrics–gynecology specialist was recommended [16]. All patients treated were included in the evaluation data.

### Intervention

The project provided resources in EDs to initiate buprenorphine/naloxone (e.g., medication supplies, provider education, order sets and patient facing documents) and referral pathways for patients [17]. There was no requirement by the health authority for EDs to participate. Spread of the program occurred through the project team networking and reaching out to sites to create awareness. The decision to implement and the timing were determined by local leaders and circumstances. Local implementation teams (including a physician champion, nursing lead, administrator, pharmacist, and social worker where available) worked to implement the program and link patients to unscheduled next-day walk-in or virtual clinic visits. Implementation and evaluation of the intervention were informed by the Consolidated Framework for Implementation Research [18]. The intervention was led by Alberta’s Emergency Strategic Clinical Network™ (ESCN) [19]. Alberta Health grant funding provided for a project manager, data analyst, and an implementation consultant/educator. The Alberta Health Services Virtual Opioid Dependency Program was included as a referral option, as it serves patients across the province [20].

Following our pilot in three sites [17], we moved to spread the program across Alberta. All sites in Alberta agreed to participate and each formed a local implementation team. To assist implementation teams, site readiness profiles were created for each site [21] (e.g., Online Resource 2). Local teams addressed contextual barriers with support of the larger project team. Locally feasible referral pathways to clinics were pre-established for each ED before program implementation. Patient access to next-day clinic visits was considered essential, as buprenorphine/naloxone is typically titrated over 2–3 days. Education for physicians, nurses, and others was conducted by local teams and five physician implementation liaisons with regional responsibilities. The definition of opioid use disorder, as given in the Diagnostic and Statistical Manual of Mental Disorders [22], was made available to ED physicians. Having a consistent program implemented across multiple sites with provincial support avoided duplication of efforts to partially alleviate staffing pressure and helped standardize care provincially. Data were collected throughout the project and results were reported at each fiscal quarter at the site level. A barriers and facilitators survey to update context assessment was run in fall 2019 [23]. Evaluation results formed the basis of tailoring the project to local sites.

### Evaluation

In this manuscript, we report results from 107 ED sites across Alberta. Twenty-four sites were able to provide data for all evaluation measures, including our primary outcomes. Table 1 provides our process, outcome and balancing measures.

**Table 1:** Measures

Type of measure	Primacy of measure	Measure description	Number of EDs reporting
Process	Primary	Number of visits where buprenorphine/naloxone was initiated	24 EDs including 13 with baseline data
Process	Secondary	Number of referrals received at participating clinics and proportion of referrals attending their appointment	NA
Process	Tertiary	Amount of buprenorphine/naloxone tablets ordered from central pharmacy by each ED	110 EDs
Outcomes	NA	Continuity of care among patients who received buprenorphine/naloxone in the ED and were discharged	24 EDs
Balancing	NA	Number and type of adverse events related to buprenorphine/naloxone	24 EDs

#### Process measures

As our primary process measure, we tracked the number of visits where buprenorphine/naloxone was dispensed. EDs tracked this for monthly reporting to our team using local electronic medical records, pyxis or manual recording (depending on what system was available in the site). Patient demographics and ED visit characteristics were extracted from the Canadian Institute for Health Information (CIHI) National Ambulatory Care Reporting System (NACRS) [24]. We recorded whether visits receiving buprenorphine/naloxone were opioid related, and the numbers of opioid-related visits in each site, by examining NACRS diagnosis fields. See Online Resource 3 for a list of International Statistical Classification of Diseases [25] diagnosis codes that were counted as opioid related. As a secondary measure, we recorded the number of referrals received at clinics from ED sites (from counts recorded manually at clinics). As a third measure, pharmacy services provided the number of buprenorphine/naloxone tablets that were ordered from pharmacy on a monthly basis.

#### Outcome measure

As our outcome measure, Alberta Pharmaceutical Information Network (PIN) data [26] were used to examine whether patients had active opioid agonist treatment prescriptions following their first relevant (index) ED visits at 30, 90 and 180 days. See Online Resource 4 for a list of Drug Identification Number [27] codes that we counted as opioid agonist treatment. For those initiated on buprenorphine/naloxone in ED, the first initiation that took place in an ED that had launched the intervention was counted as the index ED visit. For those with an opioid-related visit who were not initiated on buprenorphine/naloxone, the first opioid-related ED visit was counted as the index visit. Through quarterly reporting, we were able to assess if the intervention was enabling patients to continue with opioid agonist treatment as the program spread across sites.

#### Balancing measure

To examine potential unintended consequences of buprenorphine/naloxone initiation, the number and description of safety events related to buprenorphine/naloxone prescriptions were obtained from the provincial Reporting and Learning System for Patient Safety [28]. Particular attention was paid to the danger of “precipitated withdrawal” if buprenorphine/naloxone was provided to a patient who still had other full agonist opioids in their system [6, 29].

### Data analysis

Descriptive statistics are reported for all measures. The average monthly number of buprenorphine/naloxone initiations in each site 6 months prior to beginning the intervention and after initiating the intervention were calculated. For reporting, we categorize EDs according to provincial facility peer groups [30, 31].

For outcomes measures, only discharged patients were included, because they were eligible for both intervention components (buprenorphine/naloxone initiation and referral to a community clinic). Run charts were used to assess change in two process measures [32]. The first ten data points were used to establish a median for comparison of later outcomes to. Sites reporting zero pre-intervention buprenorphine/naloxone starts were excluded from pre–post comparisons of how many times sites initiated buprenorphine/naloxone, as this sometimes reflected reporting issues rather than initiation practice. Multivariable robust Poisson regression models were used to assess the relation of patient and ED visit characteristics to two measures of interest (receiving buprenorphine/naloxone in ED and filling a buprenorphine/naloxone prescription 30 days after initiating the medication in ED) using R version 4.1.2 [33].

## Results

### Process measures

**Fig. 1:**
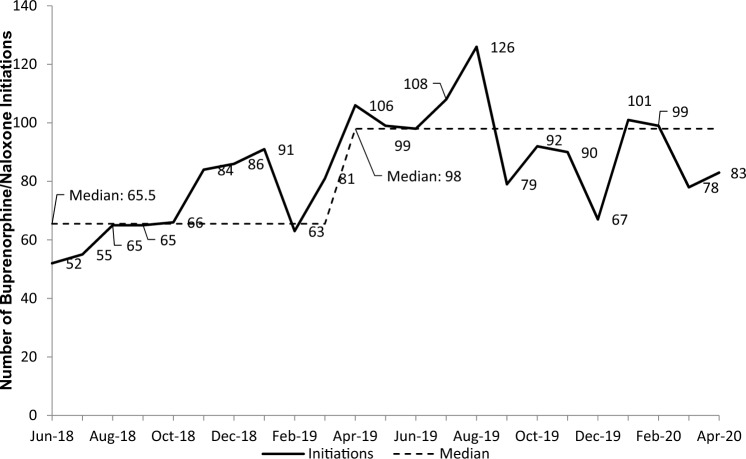
The number of buprenorphine/naloxone initiations in participating EDs after project initiation over time. May 2018 and May 2020 excluded, as our evaluation period included only 15 days of data for each

**Fig. 2:**
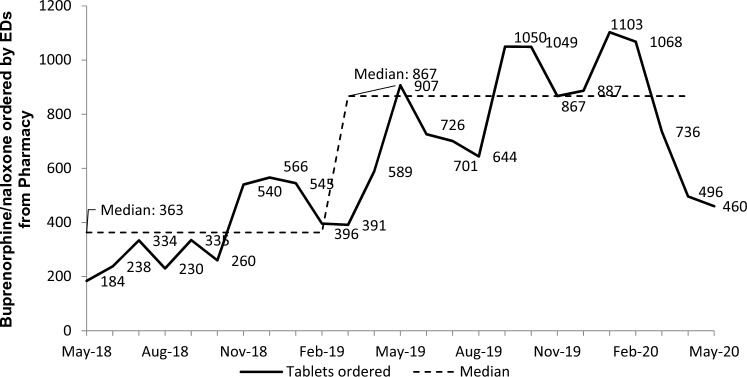
Pharmacy report overview of buprenorphine/naloxone ordering by EDs over the program evaluation period. Number of buprenorphine/naloxone tablets (2 mg/0.5 mg and 8 mg/2 mg) dispensed represents provincial total (includes all EDs in the province). Data extracted by pharmacy services

As summarized in Table 2, at the sites with pre-intervention data available, the mean number of buprenorphine/naloxone initiations per month increased post-intervention in 85% (11/13 sites) and decreased in two sites. Online resource 5 contains related site-specific data. We also include the average number of monthly opioid-related ED visits each site reported in the 6 months before and after the intervention launched at each site as intervention context. Some sites reported increased buprenorphine/naloxone initiation post-intervention in the context of fewer opioid-related visits. Figure 1 presents the number of buprenorphine/naloxone initiations in 13 EDs with baseline data. There was a shift to greater numbers of initiations later in the program, compared to the baseline median. Figure 2 shows the pharmacy report of buprenorphine/naloxone tablets stocked (ordered from the central pharmacy) within any ED in Alberta for the duration of the 2-year evaluation. There was a shift to higher numbers of tablets ordered later in the program, compared to the baseline median. Online Resource 6 presents the dates each of the 107 site teams first met to discuss our project and the date they launched the project. We show an example of an implementation checklist as Online Resource 7. A similarly detailed checklist was kept for all sites.

**Table 2:** Change in opioid-related visits and buprenorphine/naloxone initiation across 24 intervention EDs reporting buprenorphine/naloxone initiation

Facilities	Change in average monthly number of ED presentations with opioid-related diagnoses (6 months prior to the intervention compared to 6 months following intervention)	Change in average monthly number of ED presentations with buprenorphine/naloxone initiation (6 months prior to the intervention compared to 6 months following intervention)
3 tertiary^a^	3 sites ↓	2 ↓ 1 ↑
10 regional referral^b^	5 ↓ 5 ↑	5 ↑ 5 with no baseline data
3 large community^c^	2 ↓ 1 ↑	1 ↑ 2 with no baseline data
2 medium community^d^	1 no change 1 ↑	2 ↑
1 small community^e^	1 ↓	1 with no baseline data
5 ambulatory^f^ or urgent care^g^	2 ↓ 3 ↑	2 ↑ 3 with no baseline data
All 24 sites	1 no change 13 ↓ 10 ↑	2 ↓ 11 ↑ 11 with no baseline data

^a^Major hospitals providing specialized medicine

^b^Large hospitals providing access to medical specialists

^c^More than 5000 inpatients per year

^d^Less than 5000, but more than 600 inpatients per year

^e^Less than 600 inpatients per year

^f^These sites stabilize conditions that may deteriorate, have no inpatient capacity, and may require physician pre-clearance for ambulance patients

^g^These sites stabilize conditions that may deteriorate, have no inpatient capacity, and accept ambulance patients

↓—decreased, ↑—increased

Five hundred seventy-two referrals were received and recorded at clinics from participating EDs, and 271 (47%) attended their follow-up visit. Online Resource 8 shows referral locations. 84 of the follow-up visits to the VODP came from 46 hospitals not reporting buprenorphine/naloxone initiation data, showing that these sites were participating in the program to some degree.

### Outcome measure

There were 1,775 ED buprenorphine/naloxone initiations, with 1,281 of these visits pertaining to unique patients (i.e., patients could be initiated more than once). We provide a patient flow diagram as Online Resource 9. As shown in Online Resource 10, 873 (49.2%) of these patients were discharged and thus eligible for referral to clinics through our care pathway. Individuals aged 20–39 years (68%), males (59%), and patients with a CTAS score of 3 or higher comprised the largest groups of referral eligible patients. The majority of these patients were diagnosed with mental and behavioral issues related to opioids. Nine percent had a diagnosis of opioid poisoning.

Regarding continuity of care among patients who received buprenorphine/naloxone in the ED and were discharged, 671 (77%), 638 (73%), and 582 (67%) were filling a relevant prescription at 30, 90, and 180 days after their index ED visit, respectively (Online Resource 10).

Table 3 shows buprenorphine/naloxone patients aged 40–64 and 65+ years had a lower probability than those aged 20–39 years of receiving buprenorphine/naloxone in the ED. Patients in the most acute two triage categories (CTAS 1 and 2) had a lower probability of receiving buprenorphine/naloxone compared to patients whose ED visits were assessed within other triage categories. Patients with opioid poisoning had much lower probability of receiving buprenorphine/naloxone than patients with other diagnoses unrelated to opioid use.

**Table 3:** The probability of receiving buprenorphine/naloxone in ED by selected patient demographics and ED visit characteristics

Variable	Category	Relative risk (RR)	95% confidence interval	*P* value
Male compared to female		1.08	[0.96;1.23]	0.20
Age category, compared to age 20–39	10–19	0.80	[0.54;1.18]	0.26
	40–64	0.79	[0.69;0.91]	< 0.001
	65 +	0.48	[0.29;0.77]	< 0.01
CTAS score, compared to urgent	1 (resuscitation)	0.51	[0.28;0.93]	0.03
	2 (emergent)	0.74	[0.64;0.86]	< 0.001
	4 (less urgent)	1.06	[0.90;1.24]	0.49
	5 (non-urgent)	1.15	[0.88;1.50]	0.30
	Unknown	1.66	[0.47;5.88]	0.43
1st listed diagnosis, compared to other diagnoses	Mental and behavioral issues related to opioids (including withdrawal)	1.10	[0.95;1.26]	0.19
	Poisoning by opioids	0.17	[0.14;0.22]	< 0.001

Patients < 15 years of age and over > 100 years of age are excluded from all analyses, although they were eligible for bup/nal. No patients younger than 15 years received bup/nal, while six younger patients were recorded as having opioid-related ED visits. Using the < 15 year cutoff made the two groups (i.e., those receiving bup/nal versus those not receiving bup/nal) more comparable. The > 100 year cutoff was chosen to exclude patients where demographic data appeared to be erroneous (e.g., ages of 119 and 120)

Online Resource 11 shows the descriptive statistics for patients with relevant prescriptions at the 30-day mark after initiation in ED. Male patients (57%), individuals aged 40–64 years (28.5%), and patients with a CTAS score of 3 (48%) comprised the largest group of patients with an active prescription 30 days later. Table 4 shows that the probability of continuing to fill prescriptions 30 days after initiating buprenorphine/naloxone was lower for males than females, and those with opioid poisoning than patients with other diagnoses.

**Table 4:** The probability of filling an opioid agonist treatment prescription at 30 days by selected patient demographics and ED visit characteristics

Variable	Category	Relative risk (RR)	CI.95	*p* value
Male sex, compared to female		0.93	[0.86;1.00]	< 0.05
Age category, compared to age 20–39	10–19	0.88	[0.68;1.13]	0.32
	40–64	1.00	[0.92;1.09]	0.98
	65 +	0.80	[0.58;1.11]	0.19
CTAS score, compared to urgent	1 (resuscitation)	1.26	[0.89;1.78]	0.19
	2 (emergent)	1.06	[0.97;1.16]	0.22
	4 (less urgent)	1.03	[0.93;1.14]	0.55
	5 (non-urgent)	1.05	[0.89;1.23]	0.57
	Unknown	0.65	[0.25;1.67]	0.37
Diagnosis, compared to other diagnoses	Mental and behavioral issues related to opioids (including withdrawal)	0.98	[0.90;1.06]	0.57
	Poisoning by opioids	0.85	[0.73;1.00]	0.04

### Balancing measure

There were ten reported safety events. All were recorded as resulting in no apparent harm or minimal harm. These events included: medication given in the wrong amount (*n* = 7, six of which reported no apparent harm and one classified as minimal harm), medication given at the wrong time (*n* = 1, classified as minimal harm), medication not supplied when requested (*n* = 1, classified as minimal harm), and medication administered to the wrong patient (*n* = 1, classified as minimal harm).

## Discussion

### Interpretation of findings

Our findings show an increased number of buprenorphine/naloxone initiations post-intervention at most sites in comparison to baseline data. While opioid-related visits increased across Alberta over the course of our study, this was not necessarily true at the site level. Seven sites increased buprenorphine/naloxone initiation in the context of lower post-intervention opioid-related visits. We therefore believe that increased buprenorphine/naloxone initiation is related to our program rather than a simple increase in eligible patients in departments over time. A decrease in stocking of buprenorphine/naloxone tablets was observed from approximately March to May 2020. This corresponds with the early phase of pandemic measures in Alberta, with the first reported COVID-19 case in Alberta on March 5, 2020 [34].

The majority (67%) of discharged patients who received buprenorphine/naloxone in the ED had active opioid agonist treatment prescriptions 180 days after their index visit. 47% of referrals to an addiction clinic led to attendance at the first follow-up. Males were less likely to have active prescriptions at 30 days. This is concerning, as males make up a large majority of opioid-related deaths in Alberta (76.1% in 2020) [8]. Our data also show that patients diagnosed with opioid poisoning are not frequently being initiated on buprenorphine/naloxone in ED, and these patients were less likely to continue their medication prescription compared to patients with diagnoses unrelated to opioids. In the literature, there is varying expert opinion and a paucity of evidence-based recommendations on how to treat these patients. Moe and colleagues [35] recently showed that micro-dosing of buprenorphine/naloxone is a promising strategy for patients under the influence of opioids. Dosing strategies using larger than standard doses are also being investigated [36].

Reporting and Learning System for Patient Safety data from our project provide examples of the kind of safety events that can be reported in relation to ED buprenorphine/naloxone initiation. The kinds of events reported were not buprenorphine/naloxone specific (e.g., precipitated withdrawal), but errors that could occur with any medication.

### Prior studies

The demographics of the current sample were similar to those of our pilot study [17]. Most patients receiving buprenorphine/naloxone or diagnosed with an opioid-related diagnosis were aged 20–39 years. Moe and Buxton [37] highlight the importance of treating those with opioid use disorder, noting that these individuals face an early, preventable, death. The number of patients continuing to have active prescriptions at 30 and 90 days after their index ED visit were also similar to our pilot [17], suggesting the intervention continued to link patients to ongoing opioid agonist treatment as it expanded. Overall, the results of this study support findings that an ED-initiated intervention for opioid use disorder can be effective [15]. The only other jurisdictional study of buprenorphine/naloxone initiation that we are aware of in EDs also reports successful treatment initiation and follow-up [14].

### Strengths and Limitations

Our analysis offers important information on demographics and ED visit characteristics of patients who receive buprenorphine/naloxone treatment initiation in EDs and of those who continue opioid agonist treatment following ED initiation.

Our main limitations are that we relied on administrative data, could not collect all measures from all sites, and, in some sites, relied on manual reporting. The high proportion of patients receiving buprenorphine/naloxone with no opioid-related diagnosis in our data suggests that opioid use disorder may be significantly underreported in ED administrative data and confounds our comparisons between patients with opioid-related diagnoses and buprenorphine/naloxone receiving patients. Finally, Reporting and Learning System data reports only those safety events that healthcare professionals enter in the system [38]. Passive reporting systems will generally under-report safety event data.

### Clinical implications

Our program was implemented in busy EDs to create a standardized referral and opioid treatment initiation program across Alberta. We believe that the fact the program expanded across 107 EDs over 2 years, without stalling or failing to spread, is a significant accomplishment that should not be underestimated. Dong et al. [39] recently examined physician perspectives on the administration of buprenorphine/naloxone in ED. They found that physicians desired the kinds of supports (e.g., dedicated human resources) offered through our program. Other jurisdictions may benefit by adopting similar programs.

### Research implications

Studies should explore physicians’ and patients’ perspectives, identify treatment barriers, and examine ED-based opioid treatment programs using an equity lens. Studies should examine variation in physicians offering buprenorphine/naloxone (which physicians, to whom, with which effective or ineffective approaches), dosing regimens or protocols, rates of patient eligibility for opioid agonist treatment in EDs, and patient acceptance of treatment in ED. Studies should also examine treatment initiation for those presenting to ED with opioid poisoning. Such patients are at elevated risk of mortality within 1 year [40].

## Conclusions

A standardized provincial approach to initiating buprenorphine/naloxone in the ED for patients living with opioid use disorder was spread to 107 sites with dedicated program support staff and adjustment to local contexts. Similar quality improvement programs may benefit other jurisdictions.

## Supplementary Information

Below is the link to the electronic supplementary material.

Supplementary file1 (DOCX 93 KB)

## Data Availability

Access to data and code may be requested through Alberta Health Services.
