# Supplementary material for: Buprenorphine/naloxone initiation and referral as a quality improvement intervention for patients who live with opioid use disorder: quantitative evaluation of provincial spread to 107 rural and urban Alberta emergency departments
Source: CJEM. 2023 May 28;25(7):598–607. doi: 10.1007/s43678-023-00520-3 (PMC10225037; doi:10.1007/s43678-023-00520-3)
Supplement: Supplementary file 1 — Supplementary file1 (DOCX 93 KB) [file 43678_2023_520_MOESM1_ESM.docx]

**Supplementary Information**

**A multisite quantitative evaluation of buprenorphine/naloxone as an intervention in Alberta emergency departments for patients living with opioid use disorder**

**Author List:** Kayla D. Stone, PhD^1^, Ken Scott, MA, MBA^2^, Brian R. Holroyd, MD, MBA^2,3^, Eddy Lang, MD^2,4^, Karen Yee, MSc, MPH^5^, Niloofar Taghizadeh, PhD^2,4^, Janjeevan Deol, MD^3^, Kathryn Dong, MSc, MD^3^, Josh Fanaeian, MD^3^, Monty Ghosh, MBT, MSc, MD^1,6,7^, Keysha Low, RN, MHA^2^, Marshall Ross, MD^3^, Robert Tanguay, MD^1,8^, Peter Faris, PhD^5^, Nathaniel Day, MD,^9^ Patrick McLane, PhD^2,3^

## Author Affiliations:

^1^ Department of Psychiatry, University of Calgary, Canada

^2^ Emergency Strategic Clinical Network™, Alberta Health Services

^3^ Department of Emergency Medicine, University of Alberta

^4^ Department of Emergency Medicine, University of Calgary

^5^ Alberta Health Services, Data and Analytics (DIMR)

^6^ Department of Medicine, University of Calgary

^7^ Department of General Internal Medicine, University of Alberta

^8^ Department of Surgery, University of Calgary, Canada

^9^ Virtual Opioid Dependency Program, Alberta Health Services, Canada

## Corresponding author: Patrick McLane, PhD

**Online Resource 1**

**Data Collection**

Twenty-four sites were asked to provide evaluation data for all measures. See Online Resource 5 for the list of these 24 facilities and the dates they implemented the intervention. These sites were selected pragmatically with a view toward minimizing additional workload for small rural sites, while having representation from diverse areas of the province. Edmonton and Calgary “zones” cover the province’s metropolitan centers, with most of the provincial population located in these zones, and the majority of opioid-related ED visits occurring in these zones. As Edmonton and Calgary have electronic health records in EDs, all sites in these zones reported on all measures for this evaluation. However, several sites began tracking buprenorphine/naloxone initiations in ED only when they launched our project and could not provide baseline data. Seven sites reporting zero starts pre-intervention have been treated as missing baseline data, as we cannot say with certainty that these are true zeros rather than missing data. Regional hospitals in other zones (in Red Deer, Medicine Hat, Lethbridge, and Fort McMurray) were also asked to provide evaluation data. All but Fort McMurray (Northern Lights Hospital) relied on manual tracking of medication dispensing rather than electronic records. Edson and Pincher Creek were selected as smaller rural hospitals to provide data on all measures. Edson reported data electronically, beginning when our project launched, while Pincher Creek relied on manual reporting. A third rural site was asked to provide manually collected evaluation data but did not do so. Overall, pre-intervention data were available for 13 of 20 sites with electronic medical records and could not be collected from the four sites that relied on manual tracking.

Seventeen addiction clinics reported data on follow-up care during the evaluation period. Data for three clinics were excluded from analysis as these clinics ceased reporting a follow-up rate during the evaluation period. The 14 remaining clinics included those run by the health authority, as well as several independent clinics.

**Online Resource 2**

As part of the Buprenorphine/Naloxone (Suboxone^®^) Initiation in EDs project, the Emergency Strategic Clinical Network^TM^ developed a survey to administer to patient care managers, clinical nurse educators, and physician leaders in EDs across the province. Results from the survey are used to assess program readiness at each site. This document serves to highlight key results from survey respondents, and serve as a guide for site specific program implementation.

**Buprenorphine/Naloxone (Suboxone^®^) Initiation in EDs Implementation Site Readiness Profile**

| Site Readiness Score | | AHS Zone |
| --- | --- | --- |
| **1.3** | *The site readiness score is computed using responses to select questions from the survey. Responses of Strongly Agree/Agree are scored 1, and Strongly Disagree/Disagree/Neutral are scored 0. The maximum site readiness score is out of 4.*  **Provincial Average: 2.1**  **Zone Average: 2.4** | Calgary |
|  |  | Hospital/Site Name |
|  |  | Blinded |

***Survey Question: Physicians/clinicians in our emergency department see conversations about addictions and treatment as a priority.***

| Clinical Nurse Educator Response | Patient Care Manager  Response | Physician  Response |
| --- | --- | --- |
|  | Neither agree nor disagree | Neither agree nor disagree |

***Survey Question: Please list primary care programs, addictions services or community clinics that your emergency department currently has strong relationships with (leave blank if not applicable):***

| Clinical Nurse Educator Response | Patient Care Manager  Response | Physician  Response |
| --- | --- | --- |
|  | We refer to [blinded] recovery center, we make people aware of the methadone clinic. Not really strong relationships | Mental health worker and social worker in our building. We usually refer to one of them and have them discuss treatment options. If require a detox program immediately, our nursing staff will provide the available services and help facilitate transfer. |

***Survey Question: The main supports our emergency department needs to initiate Suboxone® and effectively refer patients to community follow up, excluding additional staffing, are (e.g. training, education, and processes):***

| Clinical Nurse Educator Response | Patient Care Manager  Response | Physician  Response |
| --- | --- | --- |
|  | training and education. We are minimal staffing and very busy. The teaching and time with patients would tax our current staffing. | It would be training and education |

***Survey Question: Clinicians in our emergency department communicate respectfully about patients who live with opioid use disorder when talking with other clinicians.***

| Clinical Nurse Educator Response | Patient Care Manager  Response | Physician  Response |
| --- | --- | --- |
|  | Agree | Agree |

***Survey Question: Are there patients in your emergency department who could benefit from Suboxone® treatment and referral but who do not currently receive it?***

| Clinical Nurse Educator Response | Patient Care Manager  Response | Physician  Response |
| --- | --- | --- |
|  | Yes | Yes |

***Survey Question: Why do patients not receive Suboxone® when they could benefit from it?***

| Clinical Nurse Educator Response | Patient Care Manager  Response | Physician  Response |
| --- | --- | --- |
|  | These patients are not offered Suboxone® | It is not always clear whether patients are living with OUD; Referral to treatment is not available 24/7 |

***Survey Question: The best way to engage our emergency nurses / physicians is****:*

| Clinical Nurse Educator Response | Patient Care Manager  Response | Physician  Response |
| --- | --- | --- |
|  | Educations and training. Support so that staff do not feel like this is one more task to add when they are already busy. | Offering a course or information session for the group. |

***Survey Question: Do you have any other comments?***

| Clinical Nurse Educator Response | Patient Care Manager  Response | Physician  Response |
| --- | --- | --- |
|  | The suboxone is not currently being used in any Urgent Cares. I do not know a lot about it yet. |  |

***Potential barriers and facilitators at this site:***

- Barriers to program implementation may occur when a survey response is Strongly Disagree or Disagree to relevant questions
- Facilitators to program implementation may occur when a survey response is Strongly Agree to relevant questions.

|  | **Barriers** | **Facilitators** |
| --- | --- | --- |
| Physician | None | None |
| Clinical Nurse Educators | None | None |
| Patient Care Managers | Disagreed with: Staff are available in our emergency department to support Suboxone® initiation and referral.  Disagreed with: Clinicians in our emergency department are knowledgeable about the continued cost to patients for Suboxone® and the potential need for a medication coverage plan or assistance program. | None |

**Online Resource 3**

## ICD 10 codes for opioid-related ED visits

ICD 10 codes T40XX and F11XX, for any diagnosis field (1-10):

| T40 (Poisoning by narcotics and psychodysleptics [hallucinogens] | |
| --- | --- |
| T400 | Poisoning by opium |
| T401 | Poisoning by heroin |
| T402 | Poisoning by other opioids |
| T403 | Poisoning by methadone |
| T404 | Poisoning by other synthetic narcotics |
| T406 | Poisoning by other and unspecified narcotics |
| F11 (Mental and behavioral disorders due to use of opioids) | |
| F110 | Mental and behavioural disorders due to use of opioids, acute intoxication |
| F111 | Mental and behavioural disorders due to use of opioids, harmful use |
| F112 | Mental and behavioural disorders due to use of opioids, dependence syndrome |
| F113 | Mental and behavioural disorders due to use of opioids, withdrawal state |
| F114 | Mental and behavioural disorders due to use of opioids, withdrawal state with delirium |
| F115 | Mental and behavioural disorders due to use of opioids, psychotic disorder |
| F116 | Mental and behavioural disorders due to use of opioids, amnesic syndrome |
| F117 | Mental and behavioural disorders due to use of opioids, residual and late-onset psychotic disorders |
| F118 | Mental and behavioural disorders due to use of opioids, other mental and behavioural disorders |
| F119 | Mental and behavioural disorders due to use of opioids, unspecified mental and behavioural disorders |

The following codes are excluded:

T405 – Poisoning by cocaine

T407 – Poisoning by cannabis derivatives

T408 – Poisoning by lysergide [LSD]

T409 – Poisoning by other and unspecified psychodysleptics [hallucinogens]

**Online Resource 4**

## Drug Identification Number (DIN) codes for opioid agonist therapy (OAT) prescriptions

| **DIN** | **COMPANY** | **PRODUCT** | **STRENGTH** |
| --- | --- | --- | --- |
| 02453908 | ACTAVIS PHARMA COMPANY | ACT BUPRENORPHINE/NALOXONE | 2 MG |
| 02453916 | ACTAVIS PHARMA COMPANY | ACT BUPRENORPHINE/NALOXONE | 8 MG |
| 02408090 | MYLAN PHARMACEUTICALS ULC | MYLAN-BUPRENORPHINE/NALOXONE | 2 MG |
| 02408104 | MYLAN PHARMACEUTICALS ULC | MYLAN-BUPRENORPHINE/NALOXONE | 8 MG |
| 02424851 | PHARMASCIENCE INC | PMS-BUPRENORPHINE-NALOXONE | 2 MG |
| 02424878 | PHARMASCIENCE INC | PMS-BUPRENORPHINE-NALOXONE | 8 MG |
| 02295695 | INDIVIOR UK LIMITED | SUBOXONE | 2 MG |
| 02295709 | INDIVIOR UK LIMITED | SUBOXONE | 8 MG |
| 02468085 | INDIVIOR UK LIMITED | SUBOXONE | 12 MG |
| 02468093 | INDIVIOR UK LIMITED | SUBOXONE | 16 MG |
| 02241377 | PALADIN LABS INC | METADOL | 10 MG / ML |
| 02247694 | PALADIN LABS INC | METADOL | 1 MG / ML |
| 02244290 | PALADIN LABS INC | METADOL-D | 10 MG / ML |
| 02247374 | PALADIN LABS INC | METADOL-D | 1 MG / ML |
| 02394596 | MALLINCKRODT CANADA ULC | METHADOSE | 10 MG / ML |
| 02394618 | MALLINCKRODT CANADA ULC | METHADOSE | 10 MG / ML |
| 02184435 | BGP PHARMA ULC | KADIAN | 20 MG |
| 02184443 | BGP PHARMA ULC | KADIAN | 50 MG |
| 02184451 | BGP PHARMA ULC | KADIAN | 100 MG |
| 02242163 | BGP PHARMA ULC | KADIAN | 10 MG |
| 02146126 | SANDOZ CANADA INCORPORATED | HYDROMORPHONE HP 50 | 50 MG / ML* |

Notes: *This formulation was used for injectable opioid agonist treatment during the study period.

**Online Resource 5: Facility Characteristics and Outcomes (ordered by facility type and date of intervention start)**

| Facility Name | Facility Type and Location | Intervention Start Date | ED Visits  **(2 year program)** | Mean opioid-related ED Visits 6 months  pre implementation **(ED Visits/**  **month)** | Mean opioid-related ED Visits 6 months  post implementation **(ED Visits/**  **month)** | Mean bup/nal initiations 6 months pre implementation **(ED Visits/**  **month)** | Mean bup/nal initiations 6 months post  implementation **(ED Visits/**  **month)** |  |
| --- | --- | --- | --- | --- | --- | --- | --- | --- |
| Foothills Medical Centre | Tertiary^1^ | 06-Dec-2018 | 162,497 | 58.2 | 54.7 | 6.2 | 5.5 |  |
| Royal Alexandra Hospital | Tertiary | 25-Feb-2019 | 154,583 | 151.5 | 144.0 | 35.0 | 30.5 |  |
| University of Alberta Hospital | Tertiary | 04-Mar-2019 | 130,139 | 35.3 | 28.7 | 7.0 | 10.0 |  |
| Chinook Regional Hospital | Regional Referral | 21-Feb-2019 | 112,053 | 32.3 | 23.0 | n/a | 1.2 |  |
| Grey Nuns Community Hospital | Regional Referral^2^ | 11-Jun-2018 | 144,727 | 25.0 | 26.5 | n/a | 6.2 |  |
| Medicine Hat Regional Hospital | Regional Referral | 17-Apr-2019 | 74,960 | 10.0 | 13.3 | n/a | 0.5 |  |
| Misericordia Community Hospital | Regional Referral | 05-Mar-2019 | 95,077 | 21.3 | 27.5 | n/a | 1.0 |  |
| Northern Lights Regional Health Centre | Regional Referral | 06-Jan-2020 | 129,680 | 16.2 | 12.8 | 1.8 | 2.5 |  |
| Peter Lougheed Centre | Regional Referral | 14-Mar-2019 | 158,966 | 72.2 | 71.7 | 10.8 | 17.8 |  |
| Red Deer Regional Hospital Centre | Regional Referral | 12-Dec-2018 | 112,716 | 35.7 | 21.8 | n/a | 1.7 |  |
| Rockyview General Hospital | Regional Referral | 05-Jul-2018 | 159,930 | 73.7 | 74.2 | 2.3 | 9.0 |  |
| South Health Campus | Regional Referral | 06-Dec-2018 | 138,449 | 28.0 | 28.2 | 2.8 | 5.0 |  |
| Sturgeon Community Hospital | Regional Referral | 17-Dec-2018 | 101,078 | 14.8 | 8.0 | 1.2 | 2.3 |  |
| Edson Healthcare Centre | Large Community^3^ | 01-Apr-2019 | 28,322 | 2.8 | 2.3 | n/a | 0.3 |  |
| Fort Saskatchewan Community Hospital | Large Community | 03-Jun-2019 | 35,298 | 2.5 | 4.2 | 0.3 | 1.8 |  |
| Pincher Creek Health Centre | Large Community | 15-Oct-2019 | 22,195 | 6.8 | 8.5 | n/a | 1.2 |  |
| Westview Health Centre-Stony Plain | Medium Community^4^ | 04-Jun-2019 | 51,891 | 7.3 | 7.3 | 0.3 | 1.8 |  |
| Leduc Community Hospital | Medium Community | 17-Jun-2019 | 49,964 | 6.2 | 4.0 | 0.3 | 2.0 |  |
| Devon General Hospital | Small Community^5^ | 08-Jul-2019 | 28,837 | 1.2 | 1.3 | n/a | 0.0 |  |
| Strathcona Community Hospital | Community Ambulatory Moderate^6^ | 11-Mar-2019 | 87,930 | 6.8 | 6.2 | 0.7 | 1.3 |  |
| Northeast Community Health Centre | Community Ambulatory Moderate | 15-May-2018 | 97,429 | 8.7 | 12.2 | 0.4 | 4.2 |  |
| Sheldon M. Chumir Health Centre | Urgent Care Centre^7^ | 06-Dec-2018 | 109,362 | 33.2 | 48.8 | n/a | 5.8 |  |
| South Calgary Health Centre | Urgent Care Centre | 18-Apr-2019 | 83,990 | 1.2 | 2.0 | n/a | 0.7 |  |
| East Edmonton Health Centre | Urgent Care Centre | 23-Sep-2019 | 24,543 | 3.7 | 2.5 | n/a | 0.5 |  |
| Notes: n/a = not available; there were no electronic records available to obtain pre-intervention data.   1. Major hospitals providing specialized medicine. 2. Large hospitals providing secondary access to medical specialists. 3. Less than 5000 inpatients per year. 4. Less than 5000 but more than 600 inpatients per year. 5. Less than 600 inpatients per year. 6. Stabilize conditions that may deteriorate, no inpatient capacity, require physician pre-clearance for ambulance patients. 7. Stabilize conditions that may deteriorate, no inpatient capacity, and accept ambulance patients. | | | | | | | | |

**Online Resource 6**

**Chronological Project Implementation Dates for All Sites**

| **Site** | **First Implementation Meeting Date** | **Project**  **Implementation Date** |
| --- | --- | --- |
| North East Community Health Centre | 4/23/2018 | 5/15/2018 |
| Grey Nuns Community Hospital | 4/27/2018 | 6/11/2018 |
| Rockyview General Hospital | 4/17/2018 | 7/5/2018 |
| Foothills Medical Centre | 10/9/2018 | 12/6/2018 |
| Sheldon M. Chumir Urgent Care Centre | 9/4/2018 | 12/6/2018 |
| South Health Campus | 9/27/2018 | 12/6/2018 |
| Red Deer Regional Hospital | 9/24/2018 | 12/12/2018 |
| Sturgeon Community Hospital | 10/25/2018 | 12/17/2018 |
| Chinook Regional Hospital | 12/21/2018 | 2/21/2019 |
| Royal Alexandra Hospital | 9/26/2018 | 2/25/2019 |
| University of Alberta Hospital | 11/18/2018 | 3/4/2019 |
| Misericordia Community Hospital | 11/29/2018 | 3/5/2019 |
| Strathcona Community Hospital | 10/9/2018 | 3/11/2019 |
| Peter Lougheed Centre | 11/23/2018 | 3/14/2019 |
| Edson Healthcare Centre | 1/22/2019 | 4/1/2019 |
| Sundre Hospital & Care Centre | 2/14/2019 | 4/15/2019 |
| Cardston Health Centre | 1/24/2019 | 4/15/2019 |
| Medicine Hat Regional Hospital | 3/19/2019 | 4/17/2019 |
| South Calgary Health Centre Urgent Care Centre | 2/19/2019 | 4/18/2019 |
| St. Mary's Hospital | 4/11/2019 | 5/10/2019 |
| Airdrie Community Health Centre Urgent Care Centre | 4/12/2019 | 5/17/2019 |
| Wetaskiwin Hospital & Care Centre | 4/17/2019 | 5/21/2019 |
| Rocky Mountain House Health Centre | 5/2/2019 | 5/23/2019 |
| High River General Hospital | 4/8/2019 | 6/3/2019 |
| Oilfields General Hospital | 4/16/2019 | 6/3/2019 |
| Fort Saskatchewan Community Hospital | 3/6/2019 | 6/3/2019 |
| Stony Plain Westview Health Centre | 4/10/2019 | 6/4/2019 |
| Banff Mineral Springs Hospital | 4/3/2019 | 6/5/2019 |
| Leduc Community Hospital | 3/7/2019 | 6/17/2019 |
| Rimbey Hospital & Care Centre | 5/16/2019 | 6/20/2019 |
| Cold Lake Healthcare Centre | 4/5/2019 | 7/1/2019 |
| Ponoka Hospital &Care Centre | 5/13/2019 | 7/2/2019 |
| Devon General Hospital | 5/16/2019 | 7/8/2019 |
| Drayton Valley Hospital & Care Centre | 6/17/2019 | 7/9/2019 |
| Redwater Health Centre | 4/23/2019 | 7/9/2019 |
| Bonnyville Healthcare Centre | 5/7/2019 | 7/18/2019 |
| Claresholm General Hospital | 6/25/2019 | 7/29/2019 |
| Lacombe Hospital & Care Centre | 5/23/2019 | 7/30/2019 |
| Big Country Hospital | 7/9/2019 | 8/19/2019 |
| Bassano Health Centre | 7/29/2019 | 8/29/2019 |
| Okotoks Health and Wellness Centre Urgent Care Services | 6/17/2019 | 9/3/2019 |
| Grimshaw/Berwyn & District Community Health Centre | 8/1/2019 | 9/3/2019 |
| Peace River Community Health Centre | 8/1/2019 | 9/3/2019 |
| St. Therese - St. Paul Healthcare Centre | 8/8/2019 | 9/3/2019 |
| Brooks Health Centre | 7/24/2019 | 9/3/2019 |
| Didsbury District Health Services | 6/26/2019 | 9/11/2019 |
| Canmore General Hospital | 5/9/2019 | 9/18/2019 |
| East Edmonton Health Centre | 7/5/2019 | 9/23/2019 |
| Wainwright Health Centre | 7/10/2019 | 9/25/2019 |
| Cochrane Community Health Centre Urgent Care Centre | 5/22/2019 | 10/2/2019 |
| Strathmore District Health Services | 6/21/2019 | 10/3/2019 |
| Vulcan Community Health Centre | 7/31/2019 | 10/9/2019 |
| Hinton Healthcare Centre | 8/28/2019 | 10/15/2019 |
| Crowsnest Pass Health Centre | 8/13/2019 | 10/15/2019 |
| Pincher Creek Health Centre | 8/13/2019 | 10/15/2019 |
| Hardisty Health Centre | 9/12/2019 | 10/17/2019 |
| Vermilion Health Centre | 8/7/2019 | 10/17/2019 |
| Olds Hospital & Care Centre | 9/12/2019 | 10/24/2019 |
| Raymond Health Centre | 8/14/2019 | 10/28/2019 |
| William J. Cadzow - Lac La Biche Healthcare Centre | 9/10/2019 | 10/29/2019 |
| St. Joseph's General Hospital | 7/3/2019 | 11/4/2019 |
| Two Hills Health Centre | 7/26/2019 | 11/4/2019 |
| Elk Point Healthcare Centre | 8/27/2019 | 11/4/2019 |
| Slave Lake Health Centre | 9/16/2019 | 11/4/2019 |
| Innisfail Health Centre | 9/9/2019 | 11/12/2019 |
| Tofield Health Centre | 9/12/2019 | 11/12/2019 |
| Viking Health Centre | 9/9/2019 | 11/12/2019 |
| High Prairie Health Complex | 10/8/2019 | 11/14/2019 |
| Seton - Jasper Healthcare Centre | 9/26/2019 | 11/18/2019 |
| Bow Island Health Centre | 8/8/2019 | 11/18/2019 |
| Westlock Healthcare Centre | 10/16/2019 | 11/21/2019 |
| Daysland Health Centre | 9/18/2019 | 11/25/2019 |
| Killam Health Centre | 8/21/2019 | 11/25/2019 |
| Fort Macleod Health Centre | 10/31/2019 | 11/25/2019 |
| Coronation Hospital & Care Centre | 11/25/2019 | 12/9/2019 |
| Provost Health Centre | 11/7/2019 | 12/9/2019 |
| Stettler Hospital & Care Centre | 9/9/2019 | 12/9/2019 |
| Milk River Health Centre | 10/1/2019 | 12/10/2019 |
| La Crete Community Health Centre | 11/4/2019 | 12/16/2019 |
| Northwest Health Centre | 11/4/2019 | 12/16/2019 |
| St. Theresa General Hospital | 11/4/2019 | 12/16/2019 |
| Wabasca - Desmarais Healthcare Centre | 11/13/2019 | 12/16/2019 |
| Fairview Health Complex | 9/11/2019 | 12/17/2019 |
| Whitecourt Healthcare Centre | 11/29/2019 | 12/23/2019 |
| Northern Lights Regional Health Centre | 10/7/2019 | 1/6/2020 |
| Hanna Health Centre | 11/22/2019 | 1/13/2020 |
| Three Hills Health Centre | 11/26/2019 | 1/13/2020 |
| Sacred Heart Community Health Centre | 12/11/2019 | 1/14/2020 |
| Our Lady of the Rosary Hospital | 9/25/2019 | 1/20/2020 |
| Beaverlodge Municipal Hospital | 12/10/2019 | 1/20/2020 |
| Lamont Health Care Centre | 9/11/2019 | 2/3/2020 |
| Athabasca Healthcare Centre | 10/21/2019 | 2/3/2020 |
| Barrhead Healthcare Centre | 12/10/2019 | 2/3/2020 |
| Boyle Healthcare Centre | 10/21/2019 | 2/3/2020 |
| George McDougall - Smoky Lake Healthcare Centre | 11/26/2019 | 2/3/2020 |
| Swan Hills Healthcare Centre | 12/10/2019 | 2/3/2020 |
| Taber Health Centre | 12/18/2019 | 2/3/2020 |
| Drumheller Health Centre | 10/18/2019 | 2/10/2020 |
| Central Peace Health Complex | 12/19/2019 | 2/10/2020 |
| Mayerthorpe Healthcare Centre | 11/29/2019 | 2/10/2020 |
| Coaldale Health Centre | 1/16/2020 | 2/10/2020 |
| Grande Cache Community Health Complex | 1/28/2020 | 2/18/2020 |
| Manning Community Health Centre | 10/29/2019 | 2/18/2020 |
| Fox Creek Healthcare Centre | 2/5/2020 | 3/23/2020 |
| Valleyview Health Centre | 2/5/2020 | 3/23/2020 |
| Consort Hospital & Care Centre | 1/29/2020 | 3/30/2020 |
| Sylvan Lake Community Health Centre | 3/9/2020 | 3/30/2020 |

**Online Resource 7**

**Indivdual Emergency Department Project Implementation Checklist – Example**

| Item # | Item Description | Comments | Completed |
| --- | --- | --- | --- |
| **1** | For implementation team, identify site ED physician champion(s) | Dr. Anonymous (ED Chief)  Dr. Anonymous (Physician Champion) |  |
| **2** | For implementation team, identify site ED administrative key contact (Executive, Patient Care Manager, or Unit Manager) | Anonymous (Manager)  Anonymous (Site Director) |  |
| **3** | For implementation team, identify site ED nursing  key contact (Clinical Nurse Educator or other) | Anonymous (CNE)  Anonymous (Head Nurse) |  |
| **4** | For implementation team, identify site ED pharmacy key contact | Anonymous (Pharmacist) |  |
| **5** | For implementation team, identify site ED social work key contact | N/A |  |
| **6** | ESCN Senior Provincial Director to connect with site Senior Operating Officer regarding project | Completed |  |
| **7** | ESCN to introduce awareness materials to the implementation team. Also introduce policies x3. | Awareness files sent 11-Apr-2019  Policy files sent 11-Apr-2019 |  |
| **8** | ED kick-off meeting to introduce protocol, understand concerns/risks, what is important | ED team kick-off meeting in-person [removed to preserve site anonymity].  Concerns about physician buy-in, lack of resources for follow-up in immediate community, lack of social work support in the ED. 3 First Nations groups near the hospital and some concerns regarding cell phone availability and transportation. 7-Mar-2019 phone call with Anonymized (Manager). |  |
| **9** | Establish connection(s) between ED site and community clinic(s) | 3-Apr-2019 Meeting with Dr. Nathan Day and primary care.  Anonymized (Primary Care Network pharmacist)  Anonymized (Primary Care Network Executive Director) |  |
| **10** | ESCN to work with site to help manage concerns/risks |  |  |
| **11** | Plan team roles and timing for implementation |  |  |
| **12** | Site implementation team review and confirm education plan | Clinical Nurse Educator & others’ details meeting 22-Apr-2019. |  |
| **13** | ESCN to meet with local pharmacy to confirm supply chain plan and resource support | Anonymous contacted by email. |  |
| **14** | Physician education plan roll-out | Jan Deol presenting to physicians in-person 26-Apr-2019 |  |
| **15** | Nursing and staff education plan roll-out | Go-live date [blinded] – email / 1:1 staff / voice over combo / use algorithm as high level. CIWA approach  Send presentation to Anonymous before May 10th |  |
| **16** | Patient & family education materials roll-out | Sent 22-Apr-2019 |  |
| **17** | Establish implementation team review meetings (ad hoc or interval) |  |  |
| **18** | Use of protocol for those ED physicians who are ready | Go-live date [blinded] |  |
| **19** | Use of protocol by the ED nursing and staff | Go-live date [blinded] |  |
| **20** | Project patient and administrative reporting through available data sources | Confirmed Pyxis available in the ED. Ensured medication is stocked in the ED machines for data tracking. |  |
| **21** | Monthly feedback template from the ED to the ESCN to share lessons learned |  |  |
| **22** | Ongoing support from the ESCN as needed |  |  |

**Online Resource 8**

**Locations of Clinic Referrals**

| **Location** | **Referrals from ED**  **(n=572)** | **Referrals Attending Clinic (n=271)** | **Proportion Attending** |
| --- | --- | --- | --- |
| Edmonton | 193 | 81 | 53% |
| Calgary | 236 | 89 | 38% |
| Other physical clinic location | 19 | 6 | 32% |
| Virtual Opioid Dependency Program | 124 | 95 | 83% |

**Online Resource 9**: Patients Flow Diagram


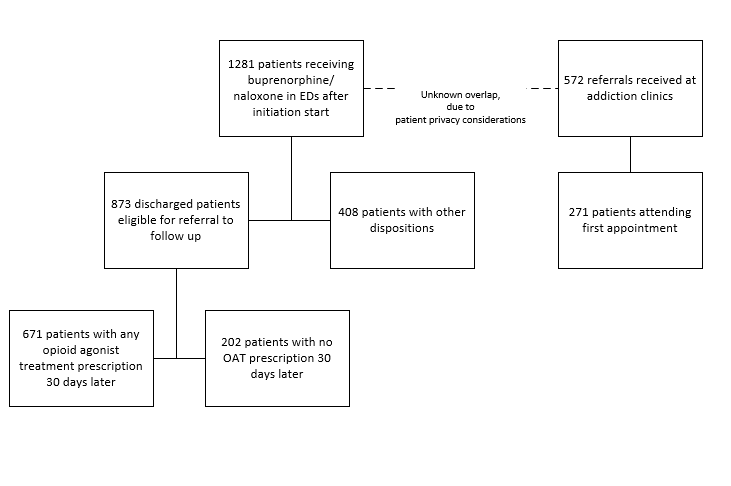


**Online Resource 10:** ED Visit Characteristics and Patient Demographics for Discharged Patients.

| **Description** | **Category** | **Receiving bup/nal** | | **Opioid-related NOT receiving bup/nal in ED** | |
| --- | --- | --- | --- | --- | --- |
|  |  | **n = 873** ^­­^ | **Row %** | **n = 4,011** | **Row %** |
| **Age Category** | 19-Oct | 22 | 2.5% | 153 | 3.8% |
|  | 20-39 | 589 | 67.5% | 2414 | 60.2% |
|  | 40-64 | 248 | 28.4% | 1321 | 32.9% |
|  | 65+ | 14 | 1.6% | 123 | 3.1% |
| **Sex ^a^** | Female | 357 | 40.9% | 1647 | 41.1% |
|  | Male | 516 | 59.1% | 2367 | 59.0% |
| **CTAS score** | Resuscitation | 9 | 1.0% | 196 | 4.9% |
|  | Emergent | 215 | 24.6% | 1826 | 45.5% |
|  | Urgent | 428 | 49.0% | 1449 | 36.1% |
|  | Less Urgent | 169 | 19.4% | 436 | 10.9% |
|  | Non-Urgent | 50 | 5.7% | 102 | 2.5% |
|  | Unknown | 2 | 0.2% | 2 | 0.0% |
| **1^st^ listed Diagnosis** | Other | 241 | 27.6% | 691 | 17.2% |
|  | Mental and behavioural issues related to opioids | 554 | 63.5% | 1370 | 34.2% |
|  | Poisoning by opioids | 78 | 8.9% | 1950 | 48.6% |
| **Active opioid agonist treatment prescription** | 30 days | 671 | 76.9% | 1752 | 43.7% |
|  | 90 days | 638 | 73.1% | 1675 | 41.8% |
|  | 180 days | 582 | 66.7% | 1551 | 38.7% |
| Notes: a Three patients changed their sex status during the 2 years of the program. | | | | | |

CTAS = Canadian Triage Acuity Scale

**Online Resource 11:** Patient Demographics and ED Visit Characteristics for Discharged Patients who Received Buprenorphine/Naloxone in ED, for those With and Without Active Opioid Agonist Treatment Prescriptions 30 days later

| **Description** | **Category** | **Filling any OAT** | | **Not filling any OAT** | |
| --- | --- | --- | --- | --- | --- |
|  |  | **n = 671** | **Row %** | **n = 202** | **Row %** |
| **Sex** | Female | 286 | 42.6% | 71 | 35.1% |
|  | Male | 385 | 57.4% | 131 | 64.9% |
| **Age category** | 10-19 | 7 | 1.0% | 15 | 7.4% |
|  | 20-39 | 133 | 19.8% | 456 | 225.7% |
|  | 40-64 | 191 | 28.5% | 57 | 28.2% |
|  | 65+ | 9 | 1.3% | 5 | 2.5% |
| **CTAS Score** | Resuscitation | 8 | 1.2% | 1 | 0.5% |
|  | Emergent | 168 | 25.0% | 47 | 23.3% |
|  | Urgent | 322 | 48.0% | 106 | 52.5% |
|  | Less Urgent | 132 | 19.7% | 37 | 18.3% |
|  | Non-Urgent | 40 | 6.0% | 10 | 5.0% |
|  | Unknown | 1 | 0.1% | 1 | 0.5% |
| **First**  **Listed Diagnosis** | Other | 191 | 28.5% | 50 | 24.8% |
|  | Mental and behavioural issues related to opioids | 426 | 63.5% | 128 | 63.4% |
|  | Poisoning by opioids | 54 | 8.0% | 24 | 11.9% |
